# The Association Between SARS-CoV-2 Exposure, COVID-19 Vaccination and Psoriatic Arthritis—A Nested Case-Control Study

**DOI:** 10.3390/vaccines14040289

**Published:** 2026-03-24

**Authors:** Amir Haddad, Ella Zabari Parnis, Nili Stein, Tal Gazitt, Walid Saliba, Devy Zisman

**Affiliations:** 1 Ruth and Bruce Rappaport Faculty of Medicine, Technion-Israel Institute of Technology, Haifa 3109601, Israel; amirha3@technion.ac.il (A.H.); eparnis@gmail.com (E.Z.P.); talgaz@clalit.org.il (T.G.); saliba_wa@clalit.org.il (W.S.); 2 Rheumatology Unit, Carmel Medical Center, Haifa 3436212, Israel; 3 Department of Epidemiology, Clalit Health Services, Haifa 3436212, Israel; stein_nili@clalit.org.il

**Keywords:** Psoriatic Arthritis (PsA), SARS-CoV-2, COVID-19, vaccine

## Abstract

**Background/Objective**: The emergence of the COVID-19 pandemic has raised significant concerns regarding its impact on immune-mediated diseases, particularly with respect to disease induction and exacerbation. We aimed to investigate the potential association between SARS-CoV-2 infection and COVID-19 vaccination and the development of Psoriatic Arthritis (PsA). **Methods**: A retrospective nested case-control study in a cohort of 3,122,602 adults without a diagnosis of PsA was conducted using a database of a large health care provider. Newly diagnosed patients with PsA aged 18 years and older were identified from the database between 1 January 2021 and 30 June 2022 and were matched by age and sex to 10 non-PsA controls. Patients were tracked to assess their exposure to SARS-CoV-2 within six months prior to diagnosis (inception date). The primary outcome of exposure to SARS-CoV-2 was compared in the cases and controls. Univariate and multivariate conditional logistic regression analyses were performed, adjusting for Body Mass Index (BMI), smoking, socioeconomic status (SES), the Charlson comorbidity index, ethnicity, psoriasis and COVID-19 vaccination status within six months. **Results**: Overall, 718 patients had a new diagnosis of PsA and were matched with 7180 controls. SARS-CoV-2 exposure among PsA cases was (N = 88/718, 12.3%) compared to controls (N = 755/7180, 10.5%), the difference was not statistically significant (*p* = 0.115). No statistically significant association was found between SARS-CoV-2 infection and PsA development after adjusting for all confounders (OR = 1.08, 95% CI [0.76–1.54], *p* = 0.652). COVID-19 vaccination was also not associated with PsA development (OR = 1.10, 95% CI [0.86–1.41], *p* = 0.45). **Conclusions**: This study found no statistically significant association between SARS-CoV-2 exposure or COVID-19 vaccination and PsA development within six months post-exposure; however, small differences cannot be excluded.

## 1. Introduction

Psoriatic Arthritis (PsA) is a complex immune-mediated disease characterized by symptoms of both psoriasis and arthritis, affecting approximately 0.1% to 1% of the global population [1].

The emergence of the COVID-19 pandemic, caused by the SARS-CoV-2 virus, has raised significant concerns regarding its impact on immune-mediated diseases, particularly with respect to disease induction and exacerbation as well as vaccination [2].

SARS-CoV-2 infection might directly exacerbate PsA symptoms through activation of Toll-like receptors and subsequent inflammatory pathways involving dendritic cells and type I interferon responses in the entheses [3]. Moreover, the interaction between SARS-CoV-2 exposure and PsA can be partially explained through the interaction of the virus with angiotensin-converting enzyme 2 (ACE2) receptors in the synovium or psoriatic lesions, enhancing tissue susceptibility to inflammation during SARS-CoV-2 infection and facilitating the virus’s pathologic impact on these tissues [4,5]. Additionally, medications initially believed to be beneficial against COVID-19, such as hydroxychloroquine, have also been implicated in the exacerbation of PsA among patients, further complicating the clinical picture [6].

Recent research has highlighted potential exacerbations of PsA due to COVID-19, with studies noting increased incidence and flare-ups following SARS-CoV-2 infection [7]. There have also been several case series and reports supporting an association between new-onset autoimmune diseases or a flare-up of preexisting autoimmune conditions following COVID-19 vaccination [8]. Messenger RNA (mRNA) vaccines, such as the Pfizer–BioNTech BNT162b2 vaccine, deliver lipid nanoparticle-encapsulated mRNA encoding the SARS-CoV-2 spike protein. Following cellular uptake, host cells transiently express the spike protein, which induces robust adaptive immune responses characterized by neutralizing antibodies and antigen-specific CD4^+^ and CD8^+^ T-cell activation. In addition to adaptive immunity, mRNA vaccines stimulate innate immune pathways through pattern-recognition receptors (e.g., Toll-like receptors), leading to type I interferon and cytokine production that enhance immunogenicity [2].

Theoretically, immune activation following infection or vaccination could, in susceptible individuals, contribute to autoimmunity through mechanisms such as molecular mimicry (structural similarity between viral/spike epitopes and self-antigens), bystander activation, epitope spreading, or nonspecific innate immune stimulation. The pandemic offers a unique lens to study the interplay between viral infections and vaccination and chronic immune-mediated diseases, which might be crucial for understanding disease pathogenesis in PsA.

This study aimed to assess whether exposure to SARS-CoV-2 and COVID-19 vaccination is associated with an increased risk of developing early PsA within six months following exposure.

## 2. Materials and Methods

This study was designed to explore the potential association between SARS-CoV-2 infection and the development of PsA. Data for this study were sourced from the Clalit Health Services (CHS) database, the largest medical databases in Israel, encompassing about 4.9 million enrollees. The study is reported in accordance with the STROBE guidelines for observational studies (see Supplementary Table S1).

### 2.1. Population

The study population was based on a cohort that consisted all of the CHS adult members ages 18 years or older as of 1 January 2021. Patients with a prior diagnosis of PsA were excluded from the analysis to ensure that only new-onset cases of PsA were evaluated. All cohort members were followed from 1 January 2021 until the diagnosis of new-onset PsA, death or end of follow-up on 30 June 2022, whichever came first. We have limited the analysis to the start date of 1 January 2021 in which the Pfizer mRNA COVID-19 vaccine was highly and the predominantly available, to avoid the confounding effect of vaccination. Vaccination was reported in the database and was analyzed as binary exposure, regardless of the number of doses or type of vaccine received.

### 2.2. Cases and Controls

All Individuals newly diagnosed with PsA during the study period were identified as the cases. The date of a new diagnosis of PsA was defined as the index date. We used a density-based sampling method to select controls from the source population. Individuals in the cohort who did not develop PsA and were alive on each case’s index date were identified. For each PsA case, 10 controls matched by age and sex were selected.

### 2.3. Data Collection

Data was extracted from the CHS database, which provided detailed demographic, clinical, and socioeconomic information for each patient. The key variables collected included:

Demographic variables: Age, sex, ethnicity (Jewish or Arab), and socioeconomic status (SES). SES was determined based on neighborhood socioeconomic scores provided by the Israeli Central Bureau of Statistics and was categorized as low, middle, or high.

Clinical variables: SARS-CoV-2 infection, defined by a positive polymerase chain reaction (PCR) test documented prior to the index date. Variant-level data were not available in the database. The data were analyzed by 3 different proposed time intervals from the index date: <14 days, 14 days to 3 months and 14 days to 6 months. Any history of COVID-19 vaccination within the same time period. Vaccination was analyzed as binary exposure (any dose) within the window, regardless of the dose number and type). Other clinical variables included Body Mass Index (BMI) categorized into four groups: underweight (<18.5), normal weight (18.5–25), overweight (25–30), and obese (>30), smoking status, Charlson comorbidity index (CCI) and diagnosis of preexisting psoriasis.

Diagnoses are captured in the registry by diagnosis-specific algorithms, employing International Classification of Diseases Ninth revision (ICD-9) code reading. The CHS database has been instrumental in various epidemiological studies, including those in the field of psoriatic disease. The reliability of the diagnosis of PsA was estimated in a previous study and found to be high with a positive predictive value, sensitivity, and specificity of 90.5%, 88.7%, and 88.1%, respectively [9].

### 2.4. Statistical Analysis

Continuous variables were summarized using means and standard deviations (SD), and categorical variables were presented as counts and proportions.

Baseline characteristics between PsA patients and non-PsA patients were compared using conditional logistic regression. Univariate and multivariate conditional logistic regression were applied to estimate the odds ratio for the association between SARS-CoV-2 infection with new-onset PsA adjusted for demographic and clinical characteristics across the three proposed time intervals before the index date (<14 days, 14 days–3 months and 14 days–6 months). However, the data presented in this study is based on the 14 days to 6 months interval to allow for a longer observation period and to better assess causality in relation to disease pathogenesis and evolution, as a very short exposure window (<14 days) is unlikely to reflect true disease or vaccine induction, and would increase the likelihood of reverse causation and so the 14-day lower bound was selected to reduce reverse causation and diagnostic work-up bias rather than to imply a true biological induction period Of note. As incidence density sampling was applied, the estimated odds ratios approximate incidence rate ratios. Missing values in the categorical covariates, BMI and SES (population sector), were handled by including a separate category for missing values. We have performed an evaluation of our conditional logistic regression model to ensure the validity and robustness of our findings. Multicollinearity was assessed using Variance Inflation Factors (VIF) for all covariates, the maximum value was 1.5, indicating no multicollinearity. We also evaluated potential influential observations using DFBETA for the COVID exposure and Vaccine COVID exposure. Both scatter plots of DFBETAs against subject identifiers confirmed that no individual matched set had a disproportionate influence on the estimated odds ratio with all values remaining close to zero.

Given the observational design, the sample size was determined by all eligible cases during the study period. A post hoc power calculation was performed. Based on 718 cases and 7180 matched controls (1:10 ratio), and an exposure prevalence of 10.5% among controls, the study had approximately 80% power (two-sided α = 0.05) to detect an odds ratio of 1.3 or greater.

Given the limited number of prespecified primary hypotheses, formal adjustment for multiple testing was not applied.

Statistical analyses were performed using SAS version 9.4 software (Cary, NC, USA). For all analyses, *p* < 0.05 for the 2-tailed tests was considered statistically significant.

### 2.5. Ethical Approval

The study was approved by the Institutional Review Board of Carmel Medical Center, Haifa (CMC0014-14).

## 3. Results

Of the 3,122,602 enrollees, 718 newly diagnosed cases of PsA were identified and matched by age and sex to 7180 controls during the study period. Patients’ characteristics are presented in Table 1. The mean age of cases (newly onset PsA) and their matched controls was 51.3 ± 15.1 of whom 44% were males. Newly diagnosed PsA patients were more obese compared with their matched controls (33% vs. 24%, *p* < 0.001) and were more likely to be smokers (44% vs. 39%, *p* = 0.012). Infection with SARS-CoV-2 documented 14 days to 6 months prior to new diagnosis of PsA was similar between cases 88 (12.3%) and controls 775 (10.5%), *p* = 0.115. COVID-19 vaccination rate within the same time period was higher between cases 409 (57.0%) compared to the controls 3822 (53.2%), *p* = 0.022.

On univariate analysis, we compared demographic and clinical factors between individuals who developed PsA (cases) and those who did not (controls). We found no difference in SARS-CoV-2 infection in either the cases or controls when tracking the interval for SARS-CoV-2 exposure of 14 days to 6 months prior to the date of inception (OR = 1.23, 95%CI 0.95–1.60, *p* = 0.115). However, smoking, history of psoriasis, the Charlson comorbidity index and history of COVID-19 vaccination were associated with PsA as shown in Table 2. Nonetheless, on multivariate analysis and after adjusting for potential confounders, including BMI, smoking status, SES, Charlson comorbidity index, psoriasis and COVID-19 vaccination, we found no statistically significant association between SARS-CoV-2 exposure and PsA development (OR = 1.08, 95%CI [0.76–1.54], *p* = 0.65) nor between a history of COVID-19 vaccination and PsA (OR = 1.10, 95%CI [0.86–1.41], *p* = 0.45). By assuming a baseline incidence of ~23 per 100,000 individuals (calculated by dividing the number of 718 new patients by 3,122,602 enrolees), an odds ratio of 1.10 would correspond to approximately 2–3 additional PsA cases per 100,000 individuals over a similar follow-up period.

Higher BMI remained a strong predictor of PsA development, with individuals having a BMI over 30 being significantly more likely to develop PsA (OR = 1.59, 95%CI [1.21–2.08], *p*< 0.001) as was the Charlson comorbidity index (OR = 1.21, 95% CI [1.10–1.31], *p* < 0.001). Smoking was not a significant predictor of PsA development in this specific adjusted model as presented in Table 2.

## 4. Discussion

This retrospective, nested, case-control study was designed to investigate whether SARS-CoV-2 infection could act as a trigger for the development of PsA, hypothesizing that the immune dysregulation caused by SARS-CoV-2 might increase the risk of PsA. However, the findings from this study did not support this hypothesis. Instead, the analysis confirmed that other well-established factors, such as preexisting psoriasis, Body Mass Index (BMI) and Charlson comorbidity index are associated with PsA onset.

The association that was observed between COVID-19 vaccination in the univariate analysis likely reflects confounding. Specifically, individuals with psoriasis and those with a higher comorbidity burden were prioritized for early COVID-19 vaccination, which may have influenced the initial observed association. After adjustment for these factors in the multivariable analysis, the association between vaccination and PsA diagnosis was attenuated and was no longer statistically significant, suggesting that the initial finding was likely due to confounding by indication.

Of note, the wide confidence interval in the multivariate analysis suggests that our study may be underpowered to detect small effects, and so small effect sizes cannot be excluded.

The lack of a statistically significant association within 6 months of viral exposure challenges previous reports suggesting a link between SARS-CoV-2 infection and the exacerbation of immune-mediated diseases [7,10]. One possible explanation is that PsA onset, similar to psoriasis, may be influenced more by chronic, long-term inflammatory states than by acute-onset inflammation such as that triggered by viral infections activating Toll-like or ACE2 receptors in tissues such as skin and synovium in the time frame investigated in the study.

Our study finding is in keeping with a study by Stiani et al. [11], which did not find a significant increase in disease flares of inflammatory arthritis after COVID-19 vaccination. Additionally, our study is in line with other studies that show that COVID-19 vaccination is not associated with new-onset PsA [12,13] within the 14 day–6-month time frame from the date of inception. Importantly, COVID-19 vaccination is highly recommended in patients with psoriatic disease, including those with comorbidities. due to its efficacy in reducing severe disease outcomes [14,15]. In contrast, a recent systematic review and metanalysis suggested that COVID-19 may be associated with an increased risk of several autoimmune rheumatic diseases. However, these findings should be interpreted with caution due to substantial inter-study heterogeneity [16].

The significant associations found between BMI and PsA support existing knowledge about the link between metabolic health and autoimmune conditions. PsA has previously been associated with metabolic syndrome, and obesity is a known risk factor for both the onset and exacerbation of inflammatory diseases. The increased adipose tissue in individuals with high BMI likely contributes to systemic inflammation through the release of pro-inflammatory cytokines, creating a chronic inflammatory environment that could trigger or worsen PsA.

Studies investigating the relationship between smoking and PsA have reported conflicting results [17]. Our study did not show an association with smoking when adjusting to other confounders in the multivariate model. Additionally, our finding on the association of Charlson comorbidity index with PsA is in keeping with the report on the effect of multimorbidity at time of psoriasis onset on risk of developing PsA [18].

Nonetheless, while the findings of this study do not show a significant link between SARS-CoV-2 infection and PsA, they reaffirm the importance of addressing modifiable risk factors such as obesity in the management of PsA. Clinicians should continue to emphasize weight management as part of comprehensive care for patients at risk. Moreover, these lifestyle modifications may not only reduce the risk of PsA but also improve outcomes for other related comorbidities, such as cardiovascular disease and diabetes, which are commonly seen in patients with psoriatic disease.

The retrospective nature of this study presents inherent limitations. Although the CHS database is noted for the accuracy of its PsA and psoriasis diagnostic codes, the reliance on medical records for diagnosing PsA and SARS-CoV-2 infection may have introduced biases, particularly if there were misclassifications or incomplete data entries. During the study period, PCR testing in Israel was widely accessible and centrally recorded; however, asymptomatic infections and home antigen testing may not have been fully captured. Additionally, there may be other confounders (such as medication use, (concomitant biologic therapy), age and sex, disease severity data, and genetic predispositions that were not accounted for in the analysis. Potentially, patients with early undiagnosed PsA may have increased healthcare contact, leading to higher likelihood of testing or vaccination. We also acknowledge that grouping all SARS-CoV-2 infections and vaccine doses into single exposure variables may oversimplify biologically distinct immune responses. During the study period, multiple variants (Alpha, Delta, Omicron) circulated, but variant-level data were not available in the database. Although the Pfizer-BioNTech mRNA vaccine was the only one available in Israel, differences between primary and booster doses could not be fully modeled. Moreover, as the study design is limited to the period of time of up to 6 months within the inception date, the results might have been different if longer time frames have been investigated, especially in delayed mechanisms that involve molecular mimicry, epigenetic reprogramming, and chronic immune activation). The 14-day lower bound was selected to reduce reverse causation and diagnostic work-up bias rather than to imply a true biological induction period. Future research should aim to address these limitations through prospective cohort studies and more comprehensive data collection. In addition, more research is needed to examine the molecular mechanisms that differentiate PsA from other autoimmune diseases in terms of viral and vaccine-triggers of disease-onset. A focus on genetic predispositions, especially within the context of viral infections could offer a deeper understanding of why some individuals are more susceptible to developing autoimmune conditions post-exposure than others. It is also important to note that although our findings were not statistically significant, the study was powered to detect moderate effect sizes (odds ratio ≥ 1.3). Therefore, while a clinically meaningful moderate or large association is unlikely, smaller effect sizes cannot be definitively excluded, as reflected by the width of the confidence intervals.

In conclusion, while this study did not find a statistically significant association between SARS-CoV-2 infection or COVID-19 vaccination and PsA within the 6-month post-exposure period, it reinforces the importance of addressing metabolic health in managing PsA risk.

### Key Points

- This large real-world study of over 3.1 million adults found no statistically significant association between SARS-CoV-2 infection or vaccination and the development of psoriatic arthritis.
- Results remained consistent after adjustment for key confounders, including BMI, smoking, comorbidities, socioeconomic status, ethnicity, and psoriasis.
- These findings suggest that COVID-19 infection and vaccination are unlikely to increase short-term PsA risk.

## Figures and Tables

**Table 1:** Characteristics of the study population.

	Variable Value	PsA (N = 718)	Controls (N = 7180)	*p*-Value
Age (mean ± SD)		51.3 ± 15.1	51.3 ± 15.1	>0.99
Sex (males%)		319 (44.4)	3190 (44.4)	>0.99
BMI Category	<25	194 (27.0)	2528 (35.1)	<0.001
	25–30	248 (34.5)	1716 (32.2)	
	>30	233 (32.5)	2493 (23.9)	
	Missing *	43 (6.0)	630 (8.8)	
Ethnic Group	Jews	545 (75.9)	5542 (77.2)	0.43
Socioeconomic status (SES)	Low	262 (36.5)	2799 (39.0)	0.53
	Middle	288 (40.1)	2826 (39.4)	
	High	164 (22.8)	1511 (21.0)	
	Missing	4 (0.6)	44 (0.6)	
Smoking N (%)		313 (43.6)	2796 (38.9)	0.012
Charlson Comorbidity Index		1.8 ± 2.1	1.6 ± 2.0	<0.001
Psoriasis		458 (63.8)	297 (4.1)	<0.001
COVID-19 Vaccination (14 days– 6 months)		409 (57.0%)	3822 (53.2%)	0.022
SARS-CoV-2 Infection (14 days–6 months)		88 (12.3%)	755 (10.5%)	0.115

* Missing data was treated as separated category, excluding missing data yielded similar results.

**Table 2:** Univariate and Multivariable Odds Ratios (ORs) for the association of SARS-CoV-2 with new-onset PsA.

	Univariate		Multivariate	
	OR 95% CI	*p*-Value	OR 95% CI	*p*-Value
**Population sector**				
Jews	**Reference**		**Reference**	
Arabs	**1.08 (0.90–1.29)**	**0.43**	**1.10 (0.83–1.46)**	**0.509**
**Socioeconomic status (SES)**				
Low	**Reference**		**Reference**	
Middle	**1.09 (0.91–1.30)**	**0.338**	**1.12 (0.86–1.45)**	**0.392**
High	**1.16 (0.95–1.43)**	**0.155**	**1.11 (0.82–1.51)**	**0.496**
**BMI**				
<25	**Reference**		**Reference**	
25–30	**1.45 (1.18–1.78)**	**<0.001**	**1.40 (1.08–1.81)**	**0.011**
>30	**1.86 (1.51–2.29)**	**<0.001**	**1.59 (1.21–2.08)**	**<0.001**
**Smoking**				
No	**Reference**		**Reference**	
Yes	**1.23 (1.05–1.45)**	**0.012**	**1.03(0.83–1.27)**	**0.787**
**Preexisting Psoriasis**				
No	**Reference**		**Reference**	
Yes	**39.4 (31.7–49.6)**	**<0.001**	**38.5 (30.7–48.4)**	**<0.001**
**Charlson Comorbidity Index**	**1.23 (1.15–1.31)**	**<0.001**	**1.21 (1.10–1.31)**	**<0.001**
**SARS-CoV-2 infection prior 14–180 days**				
No	**Reference**		**Reference**	
Yes	**1.23 (0.95–1.60)**	**0.115**	**1.08 (0.76–1.54)**	**0.652**
**COVID-19 vaccination prior 14–180 days**				
No	**Reference**		**Reference**	
Yes	**1.24 (1.03–1.50)**	**0.022**	**1.10 (0.86–1.41)**	**0.446**

## Data Availability

The data used and/or analyzed during the present study are available from the corresponding author on reasonable request.

## Supplementary Materials

The following supporting information can be downloaded at: https://www.mdpi.com/article/10.3390/vaccines14040289/s1, Table S1: STROBE checklist with corresponding manuscript sections and line numbers [19].
