# Supplementary material for: The Association Between SARS-CoV-2 Exposure, COVID-19 Vaccination and Psoriatic Arthritis—A Nested Case-Control Study
Source: Vaccines (Basel). 2026 Mar 24;14(4):289. doi: 10.3390/vaccines14040289 (PMC13119859; doi:10.3390/vaccines14040289)
Supplement: Supplementary file 1 [file vaccines-14-00289-s001.zip › vaccines-4177999-supplementary.pdf]

Table S1: STROBE checklist with corresponding manuscript sections and line numbers [19]. Study design: Nested case-control study

Section	Item	Recommendation	Line Numbers (Approx.)
**Title/Abstract**	1	Study design stated	Lines 1–3 (Title)
	2	Informative abstract	Lines ~4–25
**Introduction**	3	Background/rationale	Lines ~26–70
	4	Objectives	Lines ~70–75
**Methods**	5	Study design	Lines ~76–85
	6	Setting (location, dates)	Lines ~76–95
	7	Participants	Lines ~96–125 (Population + Cases/Controls)
	8	Variables	Lines ~126–170
	9	Data sources/measurement	Lines ~126–180
	10	Bias	Lines ~150–180 (partially) + Discussion lines ~420–470
	11	Study size	Lines ~210–220 (power calculation paragraph)
	12	Quantitative variables	Lines ~135–150 + ~180–200
	13	Statistical methods	Lines ~170–230
**Results**	14	Participants	Lines ~231–240
	15	Descriptive data	Lines ~240–260 + Table 1
	16	Outcome data	Lines ~250–270
	17	Main results	Lines ~270–310 + Table 2
	18	Other analyses	Lines ~200–230 (model diagnostics)
**Discussion**	19	Key results	Lines ~311–330
	20	Limitations	Lines ~400–470
	21	Interpretation	Lines ~330–470
	22	Generalizability	Lines ~440–470 (implicit; could be strengthened)
**Other Information**	23	Funding	Lines ~500–505
	24	Ethics	Lines ~495–500
	25	Data availability	Lines ~505–510

[19]. von Elm, E.; Altman, D.G.; Egger, M.; Pocock, S.J.; Gøtzsche, P.C.; Vandenbroucke, J.P. The Strengthening the Reporting of Observational Studies in Epidemiology (STROBE) Statement: Guidelines for Reporting Observational Studies. *PLoS Med.* **2007**, *4*, e296. https://doi.org/10.1371/journal.pmed.0040296.
